# Gene expression profiling of trout regenerating muscle reveals common transcriptional signatures with hyperplastic growth zones of the post-embryonic myotome

**DOI:** 10.1186/s12864-016-3160-x

**Published:** 2016-10-18

**Authors:** Jerôme Montfort, Aurelie Le Cam, Jean-Charles Gabillard, Pierre-Yves Rescan

**Affiliations:** INRA, UR1037 LPGP Fish Physiology and Genomics, Campus de Beaulieu, F-35042 Rennes, France

**Keywords:** Myogenesis, Muscle growth, Muscle hyperplasia, Muscle regeneration, Gene expression, Transcriptome, Teleost

## Abstract

**Background:**

Muscle fibre hyperplasia stops in most fish when they reach approximately 50 % of their maximum body length. However, new small-diameter muscle fibres can be produced *de novo* in aged fish after muscle injury. Given that virtually nothing is known regarding the transcriptional mechanisms that regulate regenerative myogenesis in adult fish, we explored the temporal changes in gene expression during trout muscle regeneration following mechanical crushing. Then, we compared the gene transcription profiles of regenerating muscle with the previously reported gene expression signature associated with muscle fibre hyperplasia.

**Results:**

Using an Agilent-based microarray platform we conducted a time-course analysis of transcript expression in 29 month-old trout muscle before injury (time 0) and at the site of injury 1, 8, 16 and 30 days after lesions were made. We identified more than 7000 unique differentially expressed transcripts that segregated into four major clusters with distinct temporal profiles and functional categories. Functional categories related to response to wounding, response to oxidative stress, inflammatory processes and angiogenesis were inferred from the early up-regulated genes, while functions related to cell proliferation, extracellular matrix remodelling, muscle development and myofibrillogenesis were inferred from genes up-regulated 30 days post-lesion, when new small myofibres were visible at the site of injury. Remarkably, a large set of genes previously reported to be up-regulated in hyperplastic muscle growth areas was also found to be overexpressed at 30 days post-lesion, indicating that many features of the transcriptional program underlying muscle hyperplasia are reactivated when new myofibres are transiently produced during fish muscle regeneration.

**Conclusion:**

The results of the present study demonstrate a coordinated expression of functionally related genes during muscle regeneration in fish. Furthermore, this study generated a useful list of novel genes associated with muscle regeneration that will allow further investigations on the genes, pathways or biological processes involved in muscle growth and regeneration in vertebrates.

**Electronic supplementary material:**

The online version of this article (doi:10.1186/s12864-016-3160-x) contains supplementary material, which is available to authorized users.

## Background

In contrast to postnatal muscle growth in mammals, which occurs exclusively through hypertrophy (size increase) of the muscle fibres formed during development, post-hatching muscle growth in many fish species combines both hypertrophy and hyperplasia (the genesis of new myofibres) [1, 2]. Muscle fibre hyperplasia in fish occurs in two successive phases. In the first phase, which generally occurs during the larval period, new fibres are formed in a discrete, continuous layer at the surface of the primary myotome. This first phase is called stratified hyperplasia. In the second phase of hyperplasia, new fibres are formed throughout the entire myotome, producing the typical mosaic appearance observed in a muscle cross section [2, 3]. Mosaic hyperplasia which is powered by resident quiescent satellite cells scattered throughout the myotome on the surface of the myofibres, eventually stops when approximately 50 % of the maximum body length is reached [3–5]. However, using a *myog:GFP* transgenic line, we recently showed that small-diameter fluorescent myofibres can be produced *de novo* in wounded post-hyperplastic muscles of aged trout [5]. This neomyogenesis, which evokes muscle regeneration following injury in adult mammals [6, 7], indicates that the myotome of aged trout still contains myogenic cells that can be reactivated *de novo* when the microenvironment is permissive, such as after damages. The regeneration of muscle in adult fish has been rarely described [5, 8], and very little is known regarding the transcriptional networks that are activated during fish regenerative myogenesis in fish. Moreover, the relationships between molecular programs that control regenerative myogenesis and muscle hyperplasia have yet to be defined. In this study, we used Agilent-based microarray platform to conduct a time-course analysis of transcript expression in the regenerating muscle of aged trout. We also compared the gene transcription profiles of regenerating muscle with the molecular signatures associated with muscle hyperplasia which we previously defined using laser capture microdissection combined with the same Agilent-based microarray platform [9].

## Results

**Fig. 1:**
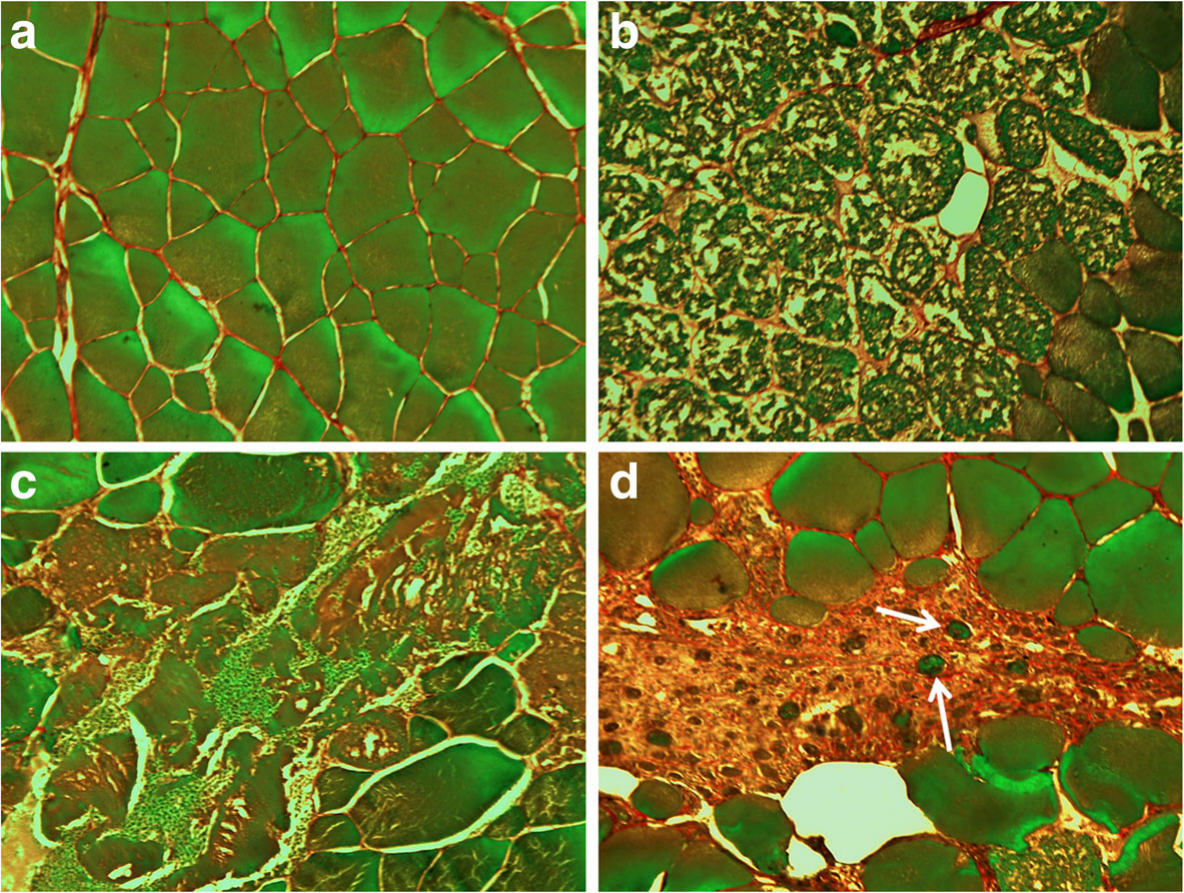
Haematoxylin and eosin histological stains of control (**a**) degenerated (**b** and **c**) and regenerating (**d**) trout muscle. Muscles were sampled at time 0 (**a**), day 1(**b**), day 16 (**c**) and day 30 (**d**) after muscle crushing. Inflammation with infiltration of inflammatory cells was seen at day 16. Small regenerating muscle fibres were seen at day 30 (arrows)

To analyse changes in gene expression profiles during muscle regeneration, we carried out, in 29 month-old trout, a time-course analysis of the transcript expression in muscle pieces excised one centimeter beneath the dorsal fin, before injury (time 0) and at the site of injury 1, 8, 16 and 30 days after lesions were made. In our experiments, we wounded the myotomal muscle in aged trout, as aged trout no longer spontaneously produce the new small myofibres that are produced by juveniles, as shown in the *myog*:GFP transgenic line [5]. At each time point, four (time 0) or five (1, 8, 16, and 30 days post lesion) distinct individuals were sampled for histological analysis and gene expression profiling. At day 1 after injury, transverse sections at the sites of lesion showed severe loss of muscle tissue. Extensive muscle damage with necrotic myofibres and inflammatory cells were visible from day 8 to day 16. At day 30 post injury, the damaged muscle regenerated new small myofibres (Fig. 1).

### Temporal transcriptome during fish muscle regeneration: Overview

**Fig. 2:**
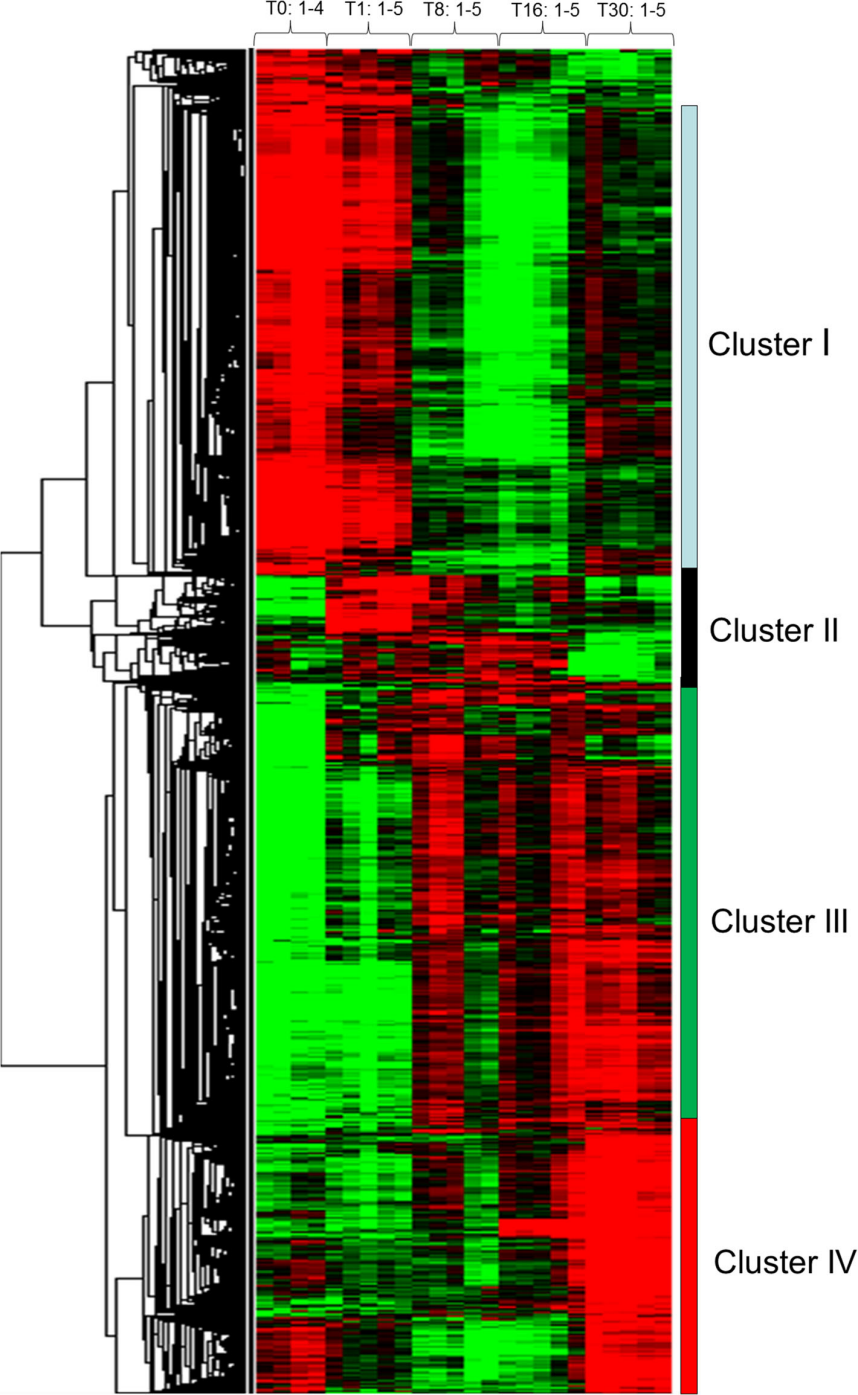
Hierarchical clustering of differentially expressed genes during muscle degeneration/regeneration. Unsupervised clustering of differentially expressed genes led to the formation of four distinct clusters (I, II, II and IV). Each row represents the temporal expression pattern of a single gene and each column corresponds to a single sample: columns 1 to 4, muscle sampled at time 0; columns 5 to 9, muscle sampled at day 1 after lesion; columns 10 to 14, muscle sampled at day 8 after lesion; columns 15 to 19, muscle sampled at day 16 after lesion; and columns 20 to 24, muscle sampled at 30 days after lesion. The expression levels are represented by coloured tags, with red representing the highest levels of expression and green representing the lowest levels of expression

An ANOVA test (BH corrected pval < 0.05) and a fold change threshold of 4 were used to define genes with expression levels that were significantly different at the different stages of sampling (e.g., T0 vs T1, T8, T16, T30; T1 vs T8, T16, T30; T8 vs T16, T30; T16 vs T30) This led to the identification of approximately 7000 unique differentially expressed genes that were then hierarchically clustered. The unsupervised clustering, which is shown in Fig. 2 and is available using heat map file (Additional file 1) and Java treeview tool (https://sourceforge.net/projects/jtreeview/files/), resulted in the formation of four major gene clusters that displayed distinct temporal profiles: cluster I was composed of genes that initially were down-regulated after muscle crushing and afterwards exhibited expression increase at day 30 post-injury; cluster II contained genes that were transiently up-regulated between 1 and 16 days post-lesion; cluster III contained genes with a sustained induction from 8 to 30 days post-lesion; and cluster IV was composed of genes specifically overexpressed at 30 days post- injury.

### Genes down-regulated after muscle injury then up-regulated afterwards

Cluster I contained 2069 unique genes which expression decreased after muscle injury and then increased at day 30 post-injury when new myofibres were formed. Contrasting to genes found in clusters III and IV, expression of genes found in cluster I was lower at day 30 post-injury than that observed in controls (non-injured muscles). 1646 genes from cluster I were eligible for analysis using DAVID software tools and were subsequently used for functional analysis. Gene Ontology of cluster I using DAVID revealed a very high enrichment in functional categories related to the generation of precursor metabolites and energy (*P* < 1.1.10^−44^, 123 genes), oxidative phosphorylation (*P* < 3.10^−16^, 41 genes), glycolysis (*P* < 6.6.10^−15^, 27 genes), myofibrils (*P* < 1.1.10^−28^, 57 genes) and muscle organ development (*P* < 1.5.10^−13^, 58 genes) (for details, see Table 1 and Additional file 2 for lists of genes that formed the major functional categories of cluster I). Specifically, regarding muscle development, it was interesting to note that cluster I contained genes encoding essential myogenic factors such as *myod1a* and *myod1b* [10] (a phylogenetic tree including the three trout paralogs of MyoD can be found in Treebase repository, see availability of supporting data), *mrf4*, *six1*, *mef2A*, *mef2C* and *nfix.*

**Table 1:** Functional categories infered from genes contained in clusters I, II, III and IV

	GO term	Cell component	P-Value	GO term	Biological process	P-Value
Cluster I	GO: 0005739	mitochondrion	1.8E-39	GO: 0006091	generation of precursor metabolites and energy	1.1E-44
	GO: 0030016	myofibrils	1.1E-28	GO: 0006936	muscle contraction	1.3E-22
	GO: 0015629	actin cytoskeleton	1.5E-14	GO: 0006006	glucose metabolic process	2.8E-20
	GO: 0016529	sarcoplasmic reticulum	2.7E-9	GO: 0006119	oxidative phosphorylation	3.0E-16
				GO: 0006096	glycolysis	6.6E-15
				GO: 0007517	muscle organ development	4.3E-13
Cluster II	none			GO: 0051254	positive regulation of RNA metabolic process	1.9E-8
				GO: 0045893	positive regulation of transcription DNA-dependent	4.3E-8
				GO: 0009891	positive regulation of biosynthetic process	2.9E-7
				GO: 0001944	vasculature development	2.5E-6
				GO: 0048514	blood vessel morphogenesis	5.7E-6
				GO: 0006979	response to oxidative stress	2.5E-5
Cluster III	GO: 0005783	endoplasmic reticulum	7.5E-14	GO: 0030029	actin filament-based process	4.2E-10
	GO: 0005764	lysosome	7.7E-12	GO: 0016192	vesicule mediated transport	8.3E-10
	GO: 0012505	endomembrane system	8.9E-9	GO: 0065003	macromolecular complex assembly	1.7E-9
	GO: 0015629	actin cytoskeleton	2.8E-8	GO: 0005996	monosaccharide metabolic process	6.9E-9
	G0: 0030529	ribonucleoprotein complex	1.0E-5	GO: 0002443	leukocyte mediated immunity	2.1E-8
				GO: 0070271	protein complex biogenesis	4.1E-8
				GO: 0002252	immune effector process	3.3E-8
				GO: 0008219	cell death	1.1E-7
				GO: 0002449	lymphocyte mediated immunity	2.4E-7
				GO: 0000398	nuclear mRNA splicing via spliceosome	4.8E-7
				GO: 0006457	protein folding	1.4E-6
				GO: 0006952	defense response	4.3E-6
				GO: 0006260	DNA replication	2.2E-5
				GO: 0006935	chemotaxis	2.7E-5
				GO: 0009611	response to wounding	7.3E-5
Cluster IV	GO: 0031012	extracellular matrix	6.5E-15	GO: 0000278	mitotic cell cycle	2.8E-15
	GO: 0030017	sarcomere	5.1E-12	GO: 0048285	organelle fission	2.6E-13
	GO: 0030016	myofibril	2.8E-11	GO: 0000279	M-phase	2.1E-12
	GO: 0015629	actin cyskeleton	4.2E-9	GO: 0030198	extracellular matrix organisation	3.6E-8
	GO: 0005581	collagen	2.3E-8	GO: 0060415	muscle tissue morphogenesis	3.6E-8
	GO: 0000793	condensed chromosome	1.1E-7	GO:0007517	muscle organ development	8.1E-8
	GO: 0000776	kinetochore	2.7E-6	GO: 0007059	chromosome segregation	1.5E-7
				GO: 0007010	cytosleleton organization	2.9E-7
				GO: 0001568	blood vessel development	8.5E-6

### Genes up-regulated early and transiently after muscle injury

Cluster II included approximately 640 unique genes with early and transient induction between 1 to 16 days post-lesion. A DAVID analysis of 531 eligible genes indicated that cluster II was highly enriched in genes involved in the positive regulation of RNA metabolic processes (*P* < 1.9.10^−8^, 42 genes), vasculature development (*P* < 2.5.10^−6^, 25 genes) and response to oxidative stress (*P* < 2.5.10^−5^, 18 genes) (for details, see Table 1 and Additional file 3 for lists of genes that formed the major functional categories of cluster II). Notably, cluster II was highly enriched for genes encoding basic leucine zipper transcription factors that bind to AP-1 DNA sites, including *ap-1/c-jun, junb, jdp2, c-fos, fosb, fra2, atf3, atf-like and atf-like3*.

### Genes with a sustained up-regulation from 8 to 30 days post-injury

Cluster III contained approximately 2300 unique genes up-regulated between 8 and 30 days post-lesion. A David analysis carried out on 1830 eligible genes indicated that this cluster was enriched in genes encoding components of the endoplasmic reticulum (*P* < 7.5.10^−14^, 182 genes) and genes involved in actin cytoskeletal rearrangements (*P* < 4.2.10^−10^, 62 genes), leukocyte-mediated immunity (*P* < 2.1.10^−8^, 30 genes), lymphocyte-mediated immunity (*P* < 2.4.10^−7^, 25 genes), immune effector processes (*P* < 3.3.10^−8^, 39 genes), defence responses (*P* < 4.3.10^−6^, 106 genes, notably including the pro-inflammatory cytokines *tnfa* and *il1b),* protein folding (*P* < 1.4.10^−6^, 43 genes) and DNA replication (*P* < 2.2.10^−5^, 42 genes) (for details see Table 1 and Additional file 4 for lists of genes that formed the major functional categories of cluster III).

### Genes up-regulated at 30 days post-injury

Cluster IV included more than 1420 unique genes specifically up-regulated 30 days post-lesion when new small muscle fibres were forming. At 30 days post-lesion, the expression levels of genes in cluster IV largely exceeded their expression levels found in non-injured muscle. The DAVID analysis of 1084 eligible genes showed that cluster IV was highly enriched in genes involved in the mitotic cell cycle (*P* < 2.8.10^−15^, 69 genes), organelle fission (*P* < 2.6.10^−13^, 49 genes) and chromosome segregation (*P* < 1.5.10^−7^, 21 genes) indicating that cell proliferation occurred only during late stages of muscle regeneration. Consistent with the production new muscle fibres observed 30 days post-lesion, cluster IV also comprised a large set of genes encoding sarcomeric proteins (*P* < 5.1.10^−12^, 30 genes including many actins, myosins, troponins and tropomyosins) and showed significant enrichment in functional categories related to muscle organ development (*P* < 8.1.10^−8^, 37 genes most notably *myod1c*, *myogenin*, *myf5*, *Tcf12* and *serum response factor*) and muscle morphogenesis (*P* < 3.6.10^−8^, 13 genes). A high enrichment of genes related to extracellular matrix (*P* < 6.5.10^−15^, 67 genes including fibronectin, laminin chains, many collagens and proteoglycans) or involved in extracellular matrix organisation (*P* < 3.6.10^−8^, 25 genes) was found in cluster IV. Finally, cluster IV was enriched in genes involved in blood vessel development (*P* < 8.5.10^−6^, 36 genes) (For details, see Table 1 and Additional file 5 for lists of genes that formed cluster IV functional categories).

### Regenerating muscle and hyperplastic growth zones share extensive common gene signature

**Fig. 3:**
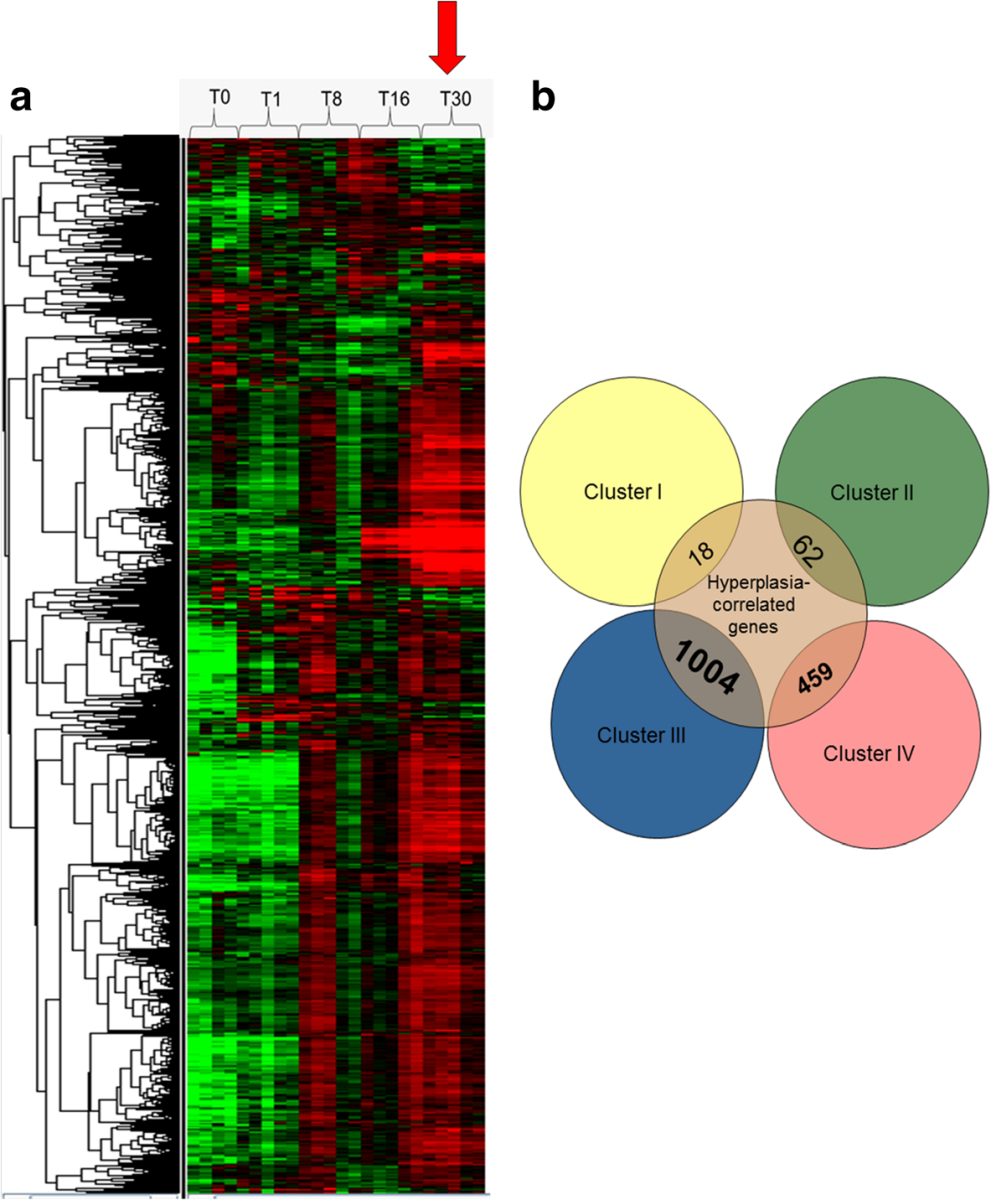
Hyperplasia-correlated genes are reactivated in regenerating muscle. **a** Supervised clustering of hyperplasia-correlated genes (as defined in [9]) during degeneration/regeneration of trout muscle: a large subset of the hyperplasia-correlated genes exhibits up-regulation during muscle regeneration, especially 30 days post-lesion (arrow), when new small myofibres are apparent. Each row represents the temporal expression pattern of a single gene and each column corresponds to a single sample, columns are as in Fig. 2. **b** Venn diagram representing the distribution of hyperplasia-correlated genes in clusters I, II, III and IV

In a previous study, using laser capture microdissection and microarray analysis, we identified 3580 unique genes overexpressed in superficial hyperplastic growth zones of the late trout embryo myotome [9]. To compare the transcriptional program of muscle regeneration and muscle hyperplasia signature, we first examined how the 3580 unique hyperplasia-correlated genes were expressed during the muscle regeneration process. We observed that most of these genes were up-regulated in regenerating muscle sampled 30 days post-injury (Fig. 3a), a stage which is concomitant with the formation of new myofibres (Fig. 1d). In line with this, a Venn diagram showed that the hyperplasia-correlated genes up-regulated in regenerating muscle were mainly within cluster III (which regrouped genes with a sustained up-regulation from 8 to 30 days post-injury) and cluster IV (which regrouped genes specifically up-regulated at 30 days post-lesion injury) (Fig. 3b). Thus, a large part of the transcriptional program underlying stratified muscle hyperplasia is reactivated during fish muscle regeneration, especially when regenerating myofibres are forming. Using a DAVID analysis, we found that the most enriched functional categories inferred from genes common to the muscle hyperplasia signature and to cluster III were related to nuclear mRNA splicing via the spliceosome (*P* < 6.1.10^−15^, 37 genes), DNA metabolic process (*P* < 1.5.10^−14^, 71 genes), DNA replication (*P* < 2.7.10^−13^, 39 genes), RNA processing (*P* < 2.2.10^−12^, 70 genes), RNP complex biogenesis (*P* < 2.7.10^−11^, 35 genes), protein folding (*P* < 1.4.10^−9^, 32 genes), macromolecular complex assembly (*P* < 2.2.10^−9^, 72 genes), and monosacharide metabolic process (*P* < 3.2.10^−7^, 32 genes) (Additional file 6). In contrast, the most enriched functional categories of the set of genes common to the muscle hyperplasia signature and to cluster IV were related to the mitotic cell cycle (*P* < 3.4.10^−26^, 52 genes), the M phase (*p* < 8.9.10^−26^, 49 genes), organelle fission (*p* < 1.4.10^−24^, 41 genes), chromosome segregation (*p* < 1.6.10^−13^, 19 genes), DNA metabolic processes (*P* < 7.2.10^−13^, 42 genes), muscle organ development (*P* < 2.10^−9^, 23 genes), muscle tissue morphogenesis (*P* < 4.1.10^−9^, 10 genes) and DNA replication (*P* < 5.2.10^−8^, 20 genes) (Additional file 7). On the other hand, the genes found in cluster III that were not up-regulated in hyperplastic growth zones displayed enrichment in functional categories related to vesicle-mediated transport, defence response, inflammatory response, response to wounding, cell death, the positive regulation of the immune system, and the regulation of cytokine production; by contrast genes found in cluster IV that were not overexpressed in hyperplastic growth zones showed enrichment in functional categories related to extracellular matrix organisation, cell adhesion, blood vessel development, the positive regulation of immune system processes and responses to wounding.

**Fig. 4:**
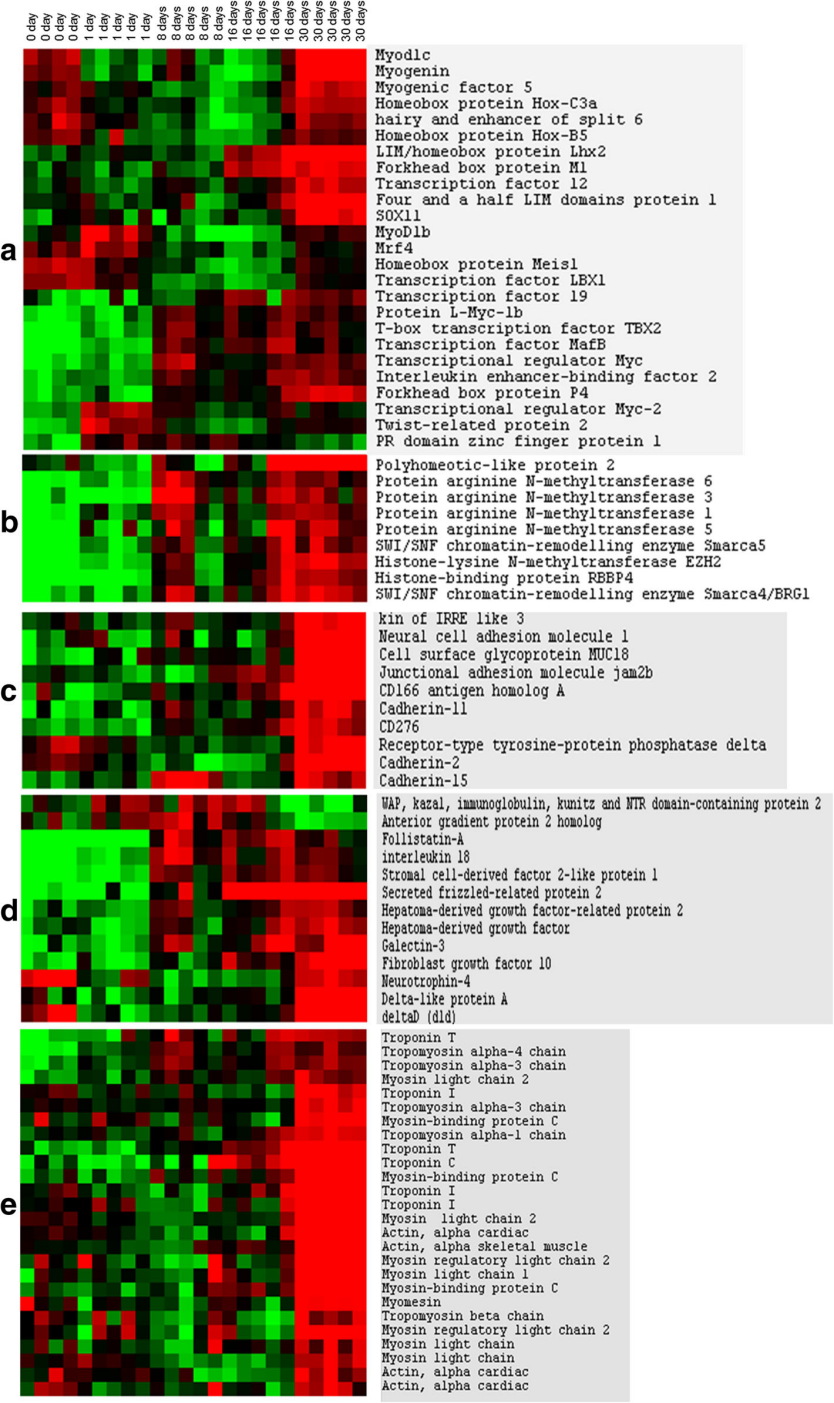
Supervised clustering of some hyperplasia-correlated genes (as defined in [9]) during degeneration/regeneration of trout muscle. **a** transcriptional regulators, **b** epigenetic transcriptional regulators, **c** immunoglobulin domain-containing membrane receptors, **d** secreted factors and **e** myofibrillar proteins. Few genes are present as multiple distinct copies resulting from paralogue retention following whole genome duplication event (WGD) that occurred at the base of the actinopterigyans or specific to the salmonid lineage. Columns are as in Fig. 2

In our previous study on the genes up-regulated in trout hyperplastic growth area, we notably focused our work on genes that were potentially involved in myogenic cell differentiation and fusion, identifying many candidate genes encoding transcriptional regulators (DNA-binding regulators and epigenetic factors), immunoglobulin (Ig) domain-containing membrane receptors and secreted factors [9]. Among the genes encoding transcriptional regulators that were up-regulated during muscle regeneration and in hyperplastic growth zones, we found canonical myogenic genes such as *myod1b*, *myod1c*, *myf5, myogenin* and *mrf4*, several homeobox-containing transcriptional regulators (*hoxb5*, *lbx1*, *meis1*, *Hoxc3* and *lhx2*), Myc paralogues (*l-myc-1b*, *myc* and *myc-2*) and various transcriptional factors including *sox11*, *tcf12*, *tcf19*, *tbx2, fhl1, mafb*, *foxm1*, *interleukin enhancer-binding factor 2*, *foxp4, twist-related protein 2*, *prdm1*, and *Hes6* (Fig. 4a). We also found several genes encoding epigenetic transcriptional regulators of the protein arginine methyltransferase (PRMT) family, such as *prmt1, prmt3, prmt5* and *prmt6*, as well as histone-lysine N-methyl transferase *ezh2*, the SWI/SNF chromatin-remodelling enzymes *smarca4/brg1* and *smarca5*, and the histone-binding protein *rbbp4* and *polyhomeotic-like protein 2* (Fig. 4b). Among the genes encoding immunoglobulin superfamily cell surface proteins that were up-regulated in both hyperplastic area and regenerating muscle, we found the promyogenic cell surface receptors *ncam1*(*cd56*), *m-cadherin* (*cadherin 15*) and *n-cadherin* (*cadherin 2*), as well as *Kin of Irre3* and *jam2b*, which are both critical for myocyte fusion in zebrafish [11, 12] (Fig. 4c). We also found the Ig superfamily members *mcam* (*cd146*), *cd 166*, *cd276* and *cadherin 11*, as well as *receptor-type tyrosine-protein phosphatases delta* (Fig. 4c). Membrane-associated proteins that were up-regulated in both regenerating and hyperplastic myogenesis also included *cleft lip and palate transmembrane protein 1-like protein*, *cklf-like marvel transmembrane domain-containing protein 7, frizzled 1* and *frizzled 7*, and *hepatocyte growth factor receptor*. The local environmental factors up-regulated in hyperplastic area and during muscle regeneration included *follistatin a* and *wfikkn2*, which both inhibit myostatin activity, as well as *secreted frizzled-related protein 2*, the notch ligands *deltad* (*dld*) and *delta-like protein a* (*dla*), *hepatoma-derived growth factor* and *hepatoma-derived growth factor 2*, *interleukin 18, neurotrophin 4, fibroblast growth factor 10, stromal cell-derived factor-2-like protein 1, galectin-3, anterior gradient protein 2* (Fig. 4d). In addition, as the same Agilent-based microarray platform was used for the gene expression profiling of both regenerating muscle and hyperplastic growth zones, we reliably calculated that nearly 80 % (26/33) of distinct myofibrillar protein encoding genes (e.g. troponins, myosin chains, myosin binding proteins, tropomyosins, alpha actins) up-regulated in injured-muscle compared to non-injured muscle were also overexpressed in hyperplastic growth zones. In Fig. 4e is shown the expression, during muscle regeneration, of myofibrillar protein encoding genes up-regulated in both hyperplastic area and regenerating muscle. Taken together, all these data indicate that a large subset of the genes highly expressed in hyperplastic growth zones and predicted to be important for myotube formation was reactivated during regenerative myogenesis, this was especially the case at 30 days post-lesion, when new myofibres formed at the site of the lesion.

### Validation of the microarray gene expression data

In order to confirm the significance of differential mRNA expression pattern observed in the microarray data, Real time PCR analysis was performed on selected genes (MyoD1a, myogenin and cadherin 15 (M-cadherin)) that exhibited distinct temporal profile during muscle regeneration. For the three genes tested, the temporal expression patterns revealed by microarray and real time PCR data were very similar (Additional file 8).

## Discussion

In this study, we explored the temporal changes in gene expression during trout muscle regeneration following mechanical wounding. Muscle regeneration has been rarely described in fish [5, 8], and virtually nothing is known regarding the genetic pathways that regulate regenerative myogenesis in this taxon. A striking feature of muscle regeneration is that muscle neofibres have not been observed in trout even 16 days post-lesion, whereas in mice, the damaged muscle is largely repaired by the same period [6, 7]. This is likely because the injured trout were maintained at a low temperature of approximately 7°C throughout the experimental period. As a result of the slowness of muscle repair in trout, regenerating myofibres were observed only in muscle sampled 30 days post-lesion. In line with this, functional categories related to myogenesis and myofibrillogenesis were found in the two clusters that contained genes that were up-regulated at 30 days during the regeneration process. The first cluster (cluster I) consisted of genes that first decreased in expression early after injury, reflecting the loss of muscle tissue caused by muscle crushes, and then increased at 30 days post-injury. This first cluster with a myogenic signature included genes encoding *mef2a* and *mef2c* which play essential roles in muscle differentiation during embryogenesis [13] and during muscle regeneration, as shown by the impairment of regenerative myogenesis in mouse resulting from the combined deletion of the *mef2* genes [7]. Interestingly, NFIX, which was also found in cluster I, has recently been shown to be required for the proper timing of muscle regeneration [14]. The second cluster (cluster IV) with a myogenic signature included genes for which the expression levels peaked only at 30 days post injury and largely exceeded their expression levels found in non-injured muscle. This cluster was highly enriched with genes involved in mitotic cell cycle, blood vessel development and extracellular matrix organization. The activation of myogenic and angiogenic programs has also been reported during exercise-induced contractile activity that leads to increased muscle mass in adult zebrafish [15]. However, genes that mediate immunity-related inflammatory processes and responses to wounding, which were concentrated in cluster III, are not activated in zebrafish hypertrophying muscle [15]. The specific inflammatory signature found in regenerating muscle is consistent with the extensive necrosis and the immune response that follow injury in vertebrates [16]. Interestingly, macrophages which are present throughout the entire regeneration process, not only have a role in the phagocytosis of damaged myofibres, but also exert effects on myogenic precursor cell proliferation (for a review see [16, 17]).

Remarkably, a large part of the genes that were up-regulated in the hyperplastic growth zones of the late embryo [9] were also strongly overexpressed in regenerating muscle sampled at 30 days post-injury, a stage at which new small myofibres were apparent. The finding of a large set of conserved genes in two forms of myogenesis suggests that this set is important for regulating post- embryonic myotube production. Among the genes that were up-regulated in both conditions were the canonical myogenic regulators (*myod*, *myf5*, *myogenin* and *mrf4*) which are indicative of satellite cell activity. In line with the overexpression of mrfs during trout muscle regeneration, it has been reported that *myf5* and *myod* are up-regulated in the regenerating muscle of zebrafish larvae [18]. Beside the canonical myogenic regulators, several transcriptional regulators with poorly documented functions in myogenesis were found; for example, we noted the up-regulation of genes encoding Sox proteins, myc paralogues and many homeodomain-containing transcriptional factors. Moreover, chromatin remodeling proteins including arginine N-methyltransferases (PRTMs) such as Prtm5, and SWI/SNF chromatin-remodelling enzymes such as Brg1/Smarca4, were up-regulated in the two forms of myogenesis. Prtm5 has a major role in controlling MRF expression and myogenesis [19], while Smarca4/Brg1 has been shown to maintain myogenic gene expression during skeletal myogenesis [20]. Notable, the proteins belonging to the Polycomb groups, which have been found to be highly expressed in hyperplastic growth zones [9], were not up-regulated during muscle regeneration. Taken together, our data suggest common and distinct epigenetic processes during muscle fibre hyperplasia and adult muscle regeneration. Many genes encoding Ig-domain-containing transmembrane proteins were also up-regulated in hyperplastic growth zones and regenerating muscle. Among these, we identified *Jam* receptor and *kin of Irre 3*, which are critical for cell fusion [11, 12], and many promyogenic cell surface receptors, such as M- and N- cadherin and NCAM, which influence cell-cell interactions during myoblast differentiation and fusion [21]. Interestingly, c-met was also up-regulated in the two forms of myogenesis. The proto-oncogene c-met, a tyrosine kinase receptor activated by hepatocyte growth factor, is involved in myoblast motility and myocyte fusion during adult skeletal muscle regeneration [22]. Among autocrine and/or paracrine factors up-regulated in hyperplastic areas and in regenerating muscle were found several regulators of the TGFβ/BMP signaling pathway such as *wfikkn2, follistatin* A and *gremlin-1*. Wfikkn2 and follistatin A both sequester myostatin, while Gremlin-1 exerts antagonistic interaction with BMP2 and BMP4. Also the common transcriptional signature included the secreted ligands *deltaD* and *delta-like protein A* that both regulate the Notch signaling on which depend satellite cell activation and adult muscle regeneration [23]. Sharp up-regulation of *sfrp2* (*secreted Frizzled-related protein 2*) was also observed in hyperplastic growth zones and during regenerative myogenesis, suggesting an active inhibition of the Wnt pathway in both situations. Although the functional significance of SFRP2 activity on myogenesis remains to be established, it is interesting to note that the up-regulation of this gene has also been reported in regenerating muscle in mice [24]. *hepatoma-derived growth factor* and *hepatoma-derived growth factor-related protein 2* were also up-regulated in the hyperplastic growth zones and during regenerative myogenesis. *hepatoma-derived growth factor* is a unique nuclear/growth factor that is involved in proliferation, differentiation and migration of various cell types and has been shown to be induced in the regenerating liver [25]. Finally, it is interesting to note that almost all myofibrillar protein encoding genes up-regulated in injured compared to non-injured muscle, were also found in the molecular signature of the hyperplastic growth zones. This further confirms the view that a large part of the transcriptional programs underlying muscle hyperplasia is reactivated in aged trout when new myofibres are transiently produced after muscle injury.

## Conclusion

In the present study, we used an Agilent-based microarray platform to carry out a time-course analysis of transcript expression during muscle regeneration in aged trout that no longer exhibit muscle hyperplasia. We identified more than 7000 unique differentially expressed transcripts that segregated into four major clusters with distinct temporal profiles and functional categories. We found that a large subset of these genes were also up-regulated in hyperplastic muscle growth zones. Notably, those genes potentially involved in differentiation and fusion of myogenic cells. The finding of a large set of conserved genes in two forms of myogenesis provides a valuable resource for further analysis of novel genes that are potentially involved in vertebrate muscle regeneration and myogenesis.

## Methods

### Animals and experimental design

Fish used in this study were reared and handled in strict accordance with French and European policies and guidelines of the INRA PEIMA Institutional Animal Care and Use Committee (B29-777-02), which specifically approved this study. Trout (*Oncorhynchus mykiss* (Walbaum) were reared in a freshwater tank (PEIMA-INRA, Sizun, France) under a natural photoperiod. The average water temperature was approximately 7 °C throughout the experimental period. Trout were 29 month old at the beginning of the experiment. Date of sampling, size and weight of the each individual female fish used in this study are reported in Additional file 9. Lesions were made in anaesthetised trout by repeatedly inserting and withdrawing a syringe needle (1.2 × 40 mm) into the trunk muscle, one centimetre beneath the dorsal fin. For sampling, the trout were killed by anaesthetic (2-phénoxyéthanol) overdose and decapitated. Lesioned site were easily locatable after injury by a lasting red colour probably resulting from blood cells infiltration. Entire blocks of fast muscle around to the site of the lesions were then excised for histological analysis and RNA extraction.

### Sample preparation and histological stains

Muscle tissues were fixed in Carnoy fixative solution for 24 h at 4°C, progressively dehydrated and embedded in paraffin. Transverse paraffin sections (10 μm thick) were stained with haematoxylin and eosin.

### Microarray slides

Microarray experiments were performed using an Agilent-based microarray platform with 8 × 60K probes per slide This platform (GEO platform record: GPL15840), which notably provides the sequence of all the oligonucleotides spotted on the slide with corresponding identifier, is based on a rainbow trout resource designed by Salem et al. [26] and was enriched with oligonucleotides designed using recent NGS data from trout (http://ngspipelines-sigenae.toulouse.inra.fr:9064/). The microarray gene annotations were reanalysed by Sigenae (Institut National de la Recherche Agronomique, Toulouse, France). Microarray data sets have been submitted to the GEO-NCBI with the accession number GSE77223.

### RNA labelling and hybridisation

Four distinct non injured trout and five distinct trout per time point post-injury were used for microarray experiments. RNA samples extracted using TRI Reagent (Sigma-Aldrich ref. T9424) were Cy3-labelled according to the manufacturer’s instructions. The labelled RNA was then fragmented in the appropriate buffer for 30 min at 60°C before dilution (v/v) in hybridisation buffer. Hybridisations were performed in a microarray hybridisation Oven overnight at 65°C, using Agilent 8x60K high-density oligonucleotide microarray slides. Following hybridisation, the slides were rinsed in gene expression wash buffers 1 and 2.

### Data acquisition and analysis

Hybridised slides were scanned with the Agilent DNA Microarray Scanner using the standard parameters for a gene expression 8x60K oligoarray (3μm and 20 bits). The data were extracted using the standard procedures contained in the Agilent Feature Extraction (FE) software version 10.7. In particular, following AGILENT instructions, a feature was validated when background substracted signal was greater than background standard deviation x2.6. The arrays were normalised (scale normalisation) and log-transformed using GeneSpring software (version 12.6.1). An ANOVA analysis (Benjamini-Hochberg (BH) corrected pval < 0.05) and a >4-fold expression change in each of the ten possible comparison were used as the criteria for defining genes as differentially expressed during muscle regeneration. For the clustering analysis, the data were median-centred and an average linkage clustering was carried out using CLUSTER software. The results were visualised using TREEVIEW [27]. GO enrichment analysis was performed using Database for annotation, Visualisation and integrated Discovery (DAVID) software tools [28, 29].

### Real-time PCR analysis

The expression of MyoD1a, myogenin and Cadherin 15 (M-cadherin) that exhibited distinct temporal expression pattern as revealed by microarray experiment, was analysed by qPCR using a real-time PCR kit incorporating a SYBR® Green fluorophore (Applied Biosystems). The relative abundance of target cDNA within the sample set was calculated from a serial dilution (1:1–1:256) (standard curve) of pool cDNA using StepOneTM Software V2.0.2 (Applied Biosystems). Subsequently, real-time PCR data were normalised by dividing the raw data by the eF1α gene expression value.

## Additional files

Additional file 1: Heat map file for Java treeview visualisation of unsupervised clustering of differentially expressed genes during regeneration. (CDT 3818 kb)
Additional file 2: Major functional categories of cluster I and lists of genes that formed them. (XLSX 31 kb)
Additional file 3: Major functional categories of cluster II and lists of genes that formed them. (XLSX 15 kb)
Additional file 4: Major functional categories of cluster III and lists of genes that formed them. (XLSX 62 kb)
Additional file 5: Major functional categories of cluster IV and lists of genes that formed them. (XLSX 25 kb)
Additional file 6: Genes and functional categories common to regenerating (cluster III) myogenesis and muscle hyperplasia. (XLSX 22 kb)
Additional file 7: Genes and functional categories common to regenerating (cluster IV) myogenesis and muscle hyperplasia. (XLSX 16 kb)
Additional file 8: (A) Relative mRNA expression levels of selected genes during muscle regeneration obtained by microarray hybridisation (left) and Q-PCR (right). Bars indicate standard error of the mean. (B) Nucleotide sequences of the PCR primers used to assay gene expression by real-time quantitative PCR. (TIF 364 kb)
Additional file 9: Weight, size and date of sampling of the animals used in the study. (XLSX 11 kb)

## Abbreviations

DAVID: Database for annotation, visualization and integrated discovery
DNA: DesoxyriboNucleic acid
GO: Gene ontology
NCBI: National Center for Biotechnology Information
RNA: RiboNucleic acid
