# Supplementary figures and images for: Gene expression profiling of trout regenerating muscle reveals common transcriptional signatures with hyperplastic growth zones of the post-embryonic myotome
Source: BMC Genomics. 2016 Oct 18;17:810. doi: 10.1186/s12864-016-3160-x (PMC5070125; doi:10.1186/s12864-016-3160-x)

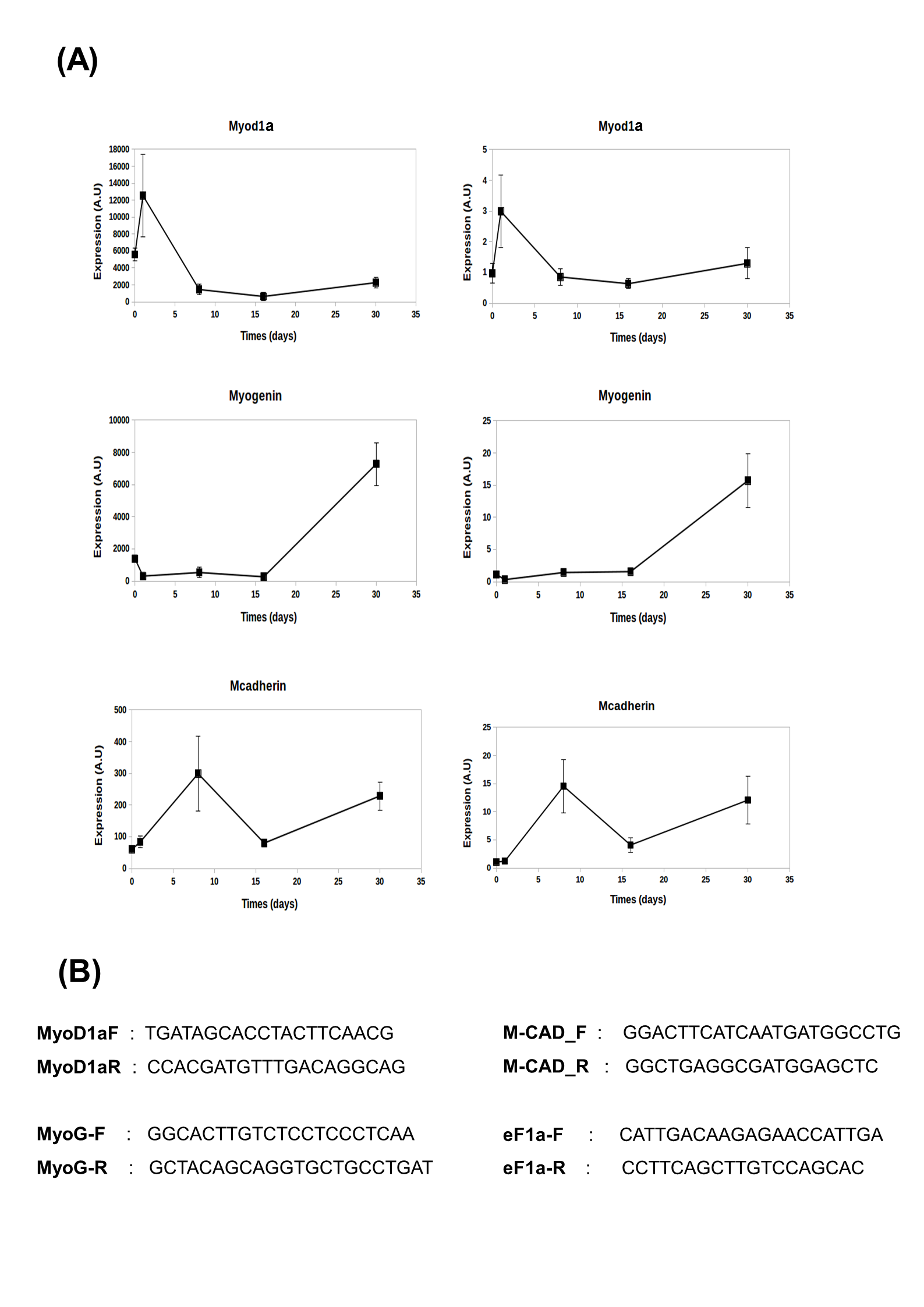

Supplement: Additional file 8: — (A) Relative mRNA expression levels of selected genes during muscle regeneration obtained by microarray hybridisation (left) and Q-PCR (right). Bars indicate standard error of the mean. (B) Nucleotide sequences of the PCR primers used to assay gene expression by real-time quantitative PCR. (TIF 364 kb) [file 12864_2016_3160_MOESM8_ESM.tif]
